# *miR-145* and *miR-133a* function as tumour suppressors and directly regulate FSCN1 expression in bladder cancer

**DOI:** 10.1038/sj.bjc.6605570

**Published:** 2010-02-16

**Authors:** T Chiyomaru, H Enokida, S Tatarano, K Kawahara, Y Uchida, K Nishiyama, L Fujimura, N Kikkawa, N Seki, M Nakagawa

**Affiliations:** 1 Department of Urology, Graduate School of Medical and Dental Sciences, Kagoshima University, Kagoshima, Japan; 2 Kawahara Nephro-urology Clinic, Kagoshima, Japan; 3 Biomedical Research Center, Chiba University, Chiba, Japan; 4 Department of Functional Genomics, Graduate School of Medicine, Chiba University, Chiba, Japan

**Keywords:** FSCN1, microRNA, *miR-145*, *miR-133a*, bladder cancer

## Abstract

**Background:**

We have recently identified down-regulated microRNAs including *miR-145* and *miR-133a* in bladder cancer (BC). The aim of this study is to determine the genes targeted by *miR-145*, which is the most down-regulated microRNA in BC.

**Methods:**

We focused on *fascin homologue 1* (*FSCN1*) from the gene expression profile in *miR-145* transfectant. The luciferase assay was used to confirm the actual binding sites of *FSCN1* mRNA. Cell viability was evaluated by cell growth, wound-healing, and matrigel invasion assays. BC specimens were subjected to immunohistochemistry of FSCN1 and *in situ* hybridisation of *miR-145*.

**Results:**

The *miR-133a* as well as *miR-145* had the target sequence of *FSCN1* mRNA by the database search, and both microRNAs repressed the mRNA and protein expression of FSCN1. The luciferase assay revealed that *miR-145* and *miR-133a* were directly bound to *FSCN1* mRNA. Cell viability was significantly inhibited in *miR-145*, *miR-133a*, and si-FSCN1 transfectants. *In situ* hybridisation revealed that *miR-145* expression was markedly repressed in the tumour lesion in which FSCN1 was strongly stained. The immunohistochemical score of FSCN1 in invasive BC (*n*=46) was significantly higher than in non-invasive BC (*n*=20) (*P*=0.0055).

**Conclusion:**

Tumour suppressive *miR-145* and *miR-133a* directly control oncogenic FSCN1 in BC.

Bladder cancer (BC) is the fifth most common cancer in the United States and the second most common cancer of the genitourinary tract (Parkin *et al*, 2005; Jemal *et al*, 2008). In Japan, the age-standardised mortality rate of BC has increased slightly since 1993 (Qiu *et al*, 2009). Currently, the standard diagnostic method depends on the use of invasive urethro-cystoscopy. Bladder tumour antigen and nuclear matrix protein-22 are available as urine markers for BC diagnostic tools. However, they are not widely used because of their low sensitivity and specificity for distinguishing BC from non-malignant diseases (van Rhijn *et al*, 2005). In the treatment of BC, morphologically similar tumours can behave differently, and it is currently not possible to identify patients who will experience tumour recurrence or disease progression (Kwak *et al*, 2004). Therefore, a new diagnostic method and treatment based on BC biology are desired.

MicroRNAs are an abundant class of small non-coding RNAs of about 22 nucleotides in length that function as negative regulators of gene expression through antisense complimentarily to specific messenger RNAs (Lagos-Quintana *et al*, 2001). Although their biological functions remain largely unknown, recent studies suggest that microRNAs contribute to the development of various cancers (Schickel *et al*, 2008). The *miR-145* and *miR-133a*/*b* have been commonly identified as down-regulated in the microRNA expression signatures of various human malignancies: head and neck carcinoma (Wang *et al*, 2008; Wong *et al*, 2008a; Childs *et al*, 2009), pancreatic ductal adenocarcinoma (Szafranska *et al*, 2007), lung cancer (Liu *et al*, 2009), breast cancer (Sempere *et al*, 2007; Wang *et al*, 2009a), gastric cancer (Takagi *et al*, 2009), colorectal cancer (Bandrés *et al*, 2006; Slaby *et al*, 2007; Schepeler *et al*, 2008; Wang *et al*, 2009b), prostate cancer (Ozen *et al*, 2008; Tong *et al*, 2009), and BC (Friedman *et al*, 2009; Lin *et al*, 2009a). In our microRNA screening test of BC, we identified a subset of seven differentially down-regulated microRNAs (*miR-145*, *miR-133a*, *miR-133b*, *miR-30a-3p*, *miR-195*, *miR-125b*, and miR-199a*) among the 156 microRNAs examined, and *miR-145* was the most down-regulated one of all (Ichimi *et al*, 2009). These studies strongly suggest that low expression levels of *miR-145* and *miR-133a*/*b* may contribute to pathogenesis and progression of human malignancies. Moreover, functional analyses of target genes, which are repressed by these microRNAs, are crucial to elucidate the mechanisms of cancer development. In this study, we performed an oligo-microarray analysis of *miR-145*-transfected BC cell lines in comparison with their parental cell lines for genome-wide screening of target genes silenced by *miR-145* in BC, and we found that *fascin homologue 1* (*FSCN1*) was the most down-regulated one among the genes.

FSCN1 is an actin-binding protein required for the formation of actin-based cell-surface protrusions and cytoplasmic bundles of microfilaments (Hashimoto *et al*, 2005). FSCN1 activity is essential to filopodial dynamics, and it has been proposed that fascin imparts rigidity to the forming filopodia to efficiently push the membrane forwards (Vignjevic *et al*, 2006). Cells with prominent cytoplasmic protrusions and actively migrating cells express high levels of FSCN1, whereas this protein is undetectable in most normal epithelial cells (Pelosi *et al*, 2003). Over-expression of FSCN1 in a variety of tumours such as lung (Pelosi *et al*, 2003), prostate (Darnel *et al*, 2009), oesophageal (Zhang *et al*, 2006), breast (Grothey *et al*, 2000), colon (Jawhari *et al*, 2003), pancreas (Maitra *et al*, 2003), ovary (Lin *et al*, 2009b), and skin cancers (Goncharuk *et al*, 2002) usually correlates with high-grade, extensive invasion, distant metastasis, and poor prognosis. However, little is known about the function of FSCN1 in BC, and it is not known whether FCSN1 expression is regulated by specific microRNAs.

We hypothesised that *miR-145* and *miR-133a*/*b* directly regulate FSCN1 and that FSCN1 has oncogenic activity in BC. We used a luciferase reporter assay to determine whether FSCN1 actually has sites targeted by *miR-145* and *miR-133a*. To investigate the functional roles of FSCN1 in BC, we performed a loss-of-function study using BC cell lines. Furthermore, we evaluated FSCN1 protein expression in clinical BC specimens by immunohistochemistry.

## Materials and methods

### Clinical samples and cell culture

The tissue specimens were from 66 BC patients who had undergone cystectomy or transurethral resection of bladder tumours at Kagoshima University Hospital between 2001 and 2005. The patient's background and clinico-pathological characteristics are summarised in Table 1. These samples were staged according to the American Joint Committee on Cancer-Union Internationale Contre le Cancer tumour-node-metastasis classification and histologically graded (Sobin and Wittekind, 2002). Normal bladder epithelia (N1 and N2) were derived from patients with non-cancerous disease and were used as the controls. Our study was approved by the Bioethics Committee of Kagoshima University; written prior informed consent and approval were given by the patients. We used three human BC cell lines; BOY was established in our laboratory from an Asian male patient aged 66 years, who had a diagnosis of stage III BC with lung metastasis (Ichimi *et al*, 2009); T24 was obtained from American Type Culture Collection; and KK47 was established in Kanazawa University and kindly provided. These cell lines were maintained in a minimum essential medium (MEM) supplemented with 10% foetal bovine serum in a humidified atmosphere of 5% CO_2_ and 95% air at 37°C.

### Mature microRNA and siRNA transfection

As earlier described (Ichimi *et al*, 2009), the transfection of BC cell lines was accomplished with RNAiMAX transfection reagent (Invitrogen, Carlisbad, CA, USA), Opti-MEM (Invitrogen) with 10 nM of mature microRNA molecules. For gain-of-function experiments, we used Pre-miR and negative-control microRNA (Applied Biosystems, Foster City, CA, USA), whereas *FSCN1* siRNA (LU-019576-00, J-019576-07, J-019576-08; Thermo Fisher Scientific, Waltham, MA, USA) and negative-control siRNA (D-001810–10; Thermo Fisher Scientific) were used for loss-of-function experiments. Cells were seeded under the following conditions: 800 000 in a 10 cm dish for protein extraction, 3000 per well in a 96-well plate for XTT assay, 200 000 per well in a 6-well plate for the wound-healing assay, and 50 000 per well in a 24-well plate for the mRNA extraction, matrigel invasion assay, and luciferase assay.

### Quantitative real-time RT–PCR

TaqMan probes and primers for FSCN1 (P/N: Hs00979631_g1; Applied Biosystems) were assay-on-demand gene expression products. All reactions were performed in duplicate and a negative-control lacking cDNA was included. Regarding the PCR conditions, we followed the manufacturer's protocol. Stem-loop RT–PCR (TaqMan MicroRNA Assays; Applied Biosystems) was used to quantitate microRNAs according to the earlier published conditions (Ichimi *et al*, 2009). For quantitative analysis of *FSCN1* mRNA and the microRNAs, *human 18s rRNA* (P/N: Hs99999901_s1; Applied Biosystems) and *RNU48* (P/N: 001006; Applied Biosystems), respectively, served as internal controls, and the delta–delta Ct methods to calculate the fold change. We used premium total RNA from normal human bladder (Clontech, Mountain View, CA, USA) as a reference.

### Gene expression analysis of BC cell lines

Total RNA was extracted by using TRIzol (Invitrogen) according to the manufacturer's protocol. The integrity of the RNA was checked with an RNA 6000 Nano Assay kit and 2100 Bioanalyzer (Agilent Technologies, Santa Clara, CA, USA). Oligo-microarray Human 44K (Agilent Technologies) was used for expression profiling in *miR-145*-transfected BC cell lines (T24 and KK47) in comparison with miR-negative-control transfectant, as described earlier (Sugimoto *et al*, 2009). Briefly, hybridisation and washing steps were performed in accordance with the manufacturer's instructions. The arrays were scanned using a Packard GSI Lumonics ScanArray 4000 (Perkin Elmer, Boston, MA, USA). The data obtained were analysed by means of DNASIS array software (Hitachi Software Engineering), which converted the signal intensity for each spot into text format. The Log2 ratios of the median subtracted background intensity were analysed. Data from each microarray study were normalised by the global normalisation method.

### Western blots

After 3 days of transfection, protein lysate (50 *μ*g) was separated by NuPAGE on 4–12% bis–tris gel (Invitrogen) and transferred into a polyvinylidene fluoride membrane. Immunoblotting was carried out with diluted (1 : 100) monoclonal FSCN1 antibody (ab49815, Abcam, Cambridge, UK) and GAPDH antibody (MAB374; Chemicon, Temecula, CA, USA). The membrane was washed and then incubated with goat anti-mouse IgG (H+L)-HRP conjugate (Bio-Rad, Hercules, CA, USA). Specific complexes were visualised with an echochemiluminescence detection system (GE Healthcare, Little Chalfont, UK).

### Cell growth, wound-healing, and matrigel invasion assays

Cell growth was determined by using an XTT assay (Roche Applied Sciences, Tokyo, Japan) that was performed according to the manufacturer's instructions. Cell migration activity was evaluated by wound-healing assay. Cells were plated in six-well dishes, and the cell monolayer was scraped using a micropipette tip. The initial gap length (0 h) and the residual gap length 24 h after wounding were calculated from Photomicrographs. A cell invasion assay was carried out using modified Boyden Chambers consisting of transwell-precoated matrigel membrane filter inserts with 8 *μ*m pores in 24-well tissue culture plates (BD Biosciences, Bedfold, MA, USA). MEM containing 10% foetal bovine serum in the lower chamber served as the chemoattractant. All experiments were performed in triplicate.

### Prediction of microRNA targets

To investigate the predicted target genes and their conserved sites in which the seed region of each microRNA binds, we used the TargetScan program (release 5.0, http://www.targetscan.org/). The sequences of the predicted mature microRNAs were confirmed by referring miRBase (release 13.0, http://microrna.sanger.ac.uk/).

### Plasmid construction and dual-luciferase assay

MicroRNA target sequences were inserted between the XhoI–PmeI restriction sites in the 3′-UTR of the hRluc gene in psiCHECK-2 vector (C8021, Promega, Madison, WI, USA). BOY cells were transfected with 5 ng of vector, 10 nM of microRNAs, and 1 *μ*l of Lipofectamine 2000 (Invitrogen) in a 100 *μ*l Opti-MEM. The activities of firefly and *Renilla* luciferases in cell lysates were determined with a dual-luciferase assay system (Promega). Normalised data were calculated as the quotient of *Renilla*/firefly-luciferase activities.

### Immunohistochemistry

The primary mouse monoclonal antibodies against FSCN1 (Abcam) were diluted by 1 : 200. The slides were treated with Biotinylated Anti-Mouse IgG (H+L) made in horse (Vector laboratories, Burlingame, CA, USA). Diaminobenzidine-hydrogen peroxide (Sigma-Aldrich, St Louis, MO, USA) was the chromogen, and the counterstaining was carried out with 0.5% haematoxylin. The positivity of endothelia and myofiblasts served as an inner positive control. The intensity of the staining was scored as negative (0), weak (1), moderate (2), or strong (3) (Ropponen *et al*, 1999). All staining scores are averages of duplicate experiments, and all samples were independently scored by two of us (TC and HE) who were blinded to the patient status.

### *In situ* hybridisation of microRNA

*In situ* hybridisation was carried out according to the manufacturer's protocol for formalin-fixed, paraffin-embedded (FFPE) tissue (Kloosterman *et al*, 2006) on human BC specimens. DIG-labelled LNA oligo-nucleotides were purchased from EXIQON (Woburm, MA, USA) and used for overnight hybridisation at 52°C. The staining was carried out as described earlier. After deparaffinisation, the specimens were subjected to proteinase K (20 Ag per ml) digestion for 20 min. The post-fixed tissues were subsequently incubated overnight with the locked nucleic acid-modified probes. For the immunodetection, tissues were incubated overnight at 4°C in anti-DIG-AP FAB fragment (Roche Applied Sciences; 1/2000). The final visualisation was carried out with NBT/BCIP (Pierce, Rockford, IL, USA).

### Statistical analysis

The relationship between two variables and the numerical values obtained by real-time RT–PCR was analysed using the Mann–Whitney *U*-test. The relationship between three variables and the numerical values was analysed using the Bonferroni-adjusted Mann–Whitney *U*-test. The analysis software was Expert StatView (version 4, SAS Institute Inc., Cary, NC, USA); for the comparison test among the three variables, a non-adjusted statistical level of significance of *P*<0.05 corresponds to a Bonferroni-adjusted level of *P*<0.0167.

## Results

### Gene expression profile identifying down-regulated genes in *miR-145* transfectant

For genome-wide screening of target genes silenced by *miR-145* in BC, we performed an oligo-microarray analysis of *miR-145*-transfected BC cell lines (T24 and KK47) in comparison with miR-negative-control transfectant. A total of 200 genes were generally down-regulated by >0.5-fold in *miR-145* transfectants compared with the control. We focused on *FSCN1* because it was listed as the top candidate in the expression profile (Table 2).

### FSCN1 as a target of post-transcriptional repression by *miR-145* and *miR-133a*

**Figure 1:**
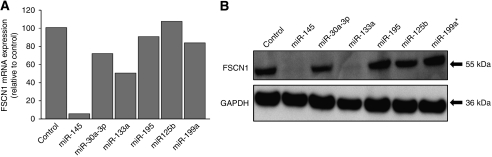
Regulation of FSCN1 expression in the down-regulated microRNA transfectants (T24). (**A**) *FSCN1* mRNA expression after 24 h transfection with 10 nM of microRNAs (*miR-145*, *miR-30a-3p*, *miR-133a*, *miR-195*, *miR-125b*, and miR-199a*). *FSCN1* mRNA expression was repressed in *miR-145* and *miR-133a* transfectants. (**B**) FSCN1 protein expression after 72 h transfection of microRNAs. Glyceraldehyde-3-phosphate dehydrogenase (GAPDH) was used as a loading control. The protein expression level of FSCN1 was also repressed in the transfectants.

**Figure 2:**
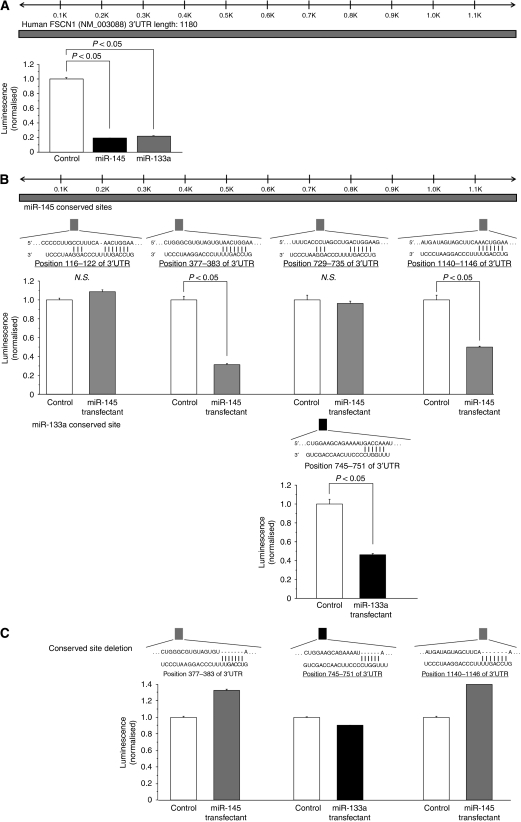
*miR-145-* and *miR-133a-*binding sites in *FSCN1* 3′-UTR. (**A**) A luciferase assay using the vector encoding full length of *FSCN1* 3′-UTR (position 51–1180). BOY cells were transfected with 5 ng vector and 10 nM microRNAs. The *Renilla* luciferase values were normalised by firefly-luciferase values. (**B**) Luciferase assays using the vectors encoding putative conserved target sites of *FSCN1* 3′-UTR identified with the TargetScan database: four conserved sites for *miR-145* and one site for *miR-133a*. (**C**) Luciferase assays using the mutated vectors in which the specific sites targeted by the microRNAs were deleted.

Among the T24 cell lines transfected with the six down-regulated microRNAs in our earlier study (Ichimi *et al*, 2009), the expression levels of *FSCN1* mRNA and its protein were markedly decreased not only in *miR-145*, but also in *miR-133a* transfectants (Figure 1A and B). We performed a luciferase assay to determine whether *FSCN1* mRNA actually has the target sites of these two microRNAs, as indicated by the TargetScan algorithm. We initially used the vector encoding full-length 3′-UTR of *FSCN1* mRNA (position 51–1180), and the luminescence intensity was significantly decreased in *miR-145* and *miR-133a* transfectants (Figure 2A). Furthermore, to determine the specific sites targeted by the two microRNAs, we constructed vectors covering four conserved sites for *miR-145* and one site for *miR-133a* (Figure 2B). The luminescence intensity was significantly decreased at the two sites targeted by *miR-145* (positions 377–383 and 1140–1146) and one site targeted by *miR-133a* (position 745–751) (Figure 2B). In addition, we constructed three mutated vectors in which the specific sites targeted by the microRNAs were deleted, and the luminescence intensity was not decreased at all by *miR-145* and *miR-133a* (Figure 2C). We did not examine *miR-133b* because it was considered to function similarly to *miR-133a*; these microRNAs have very similar sequences (*miR-133a*: UUGGUCCCCUUCAACCAGCUGU, *miR-133b*: UUGGUCCCCUUCAACCAGCUA) and have a common sequence for binding to *FSCN1* mRNA (UUGGUC) (Figure 2B).

### Effect of *miR-145* and *miR-133a* transfection on cell growth, invasion, and migration activity in BC cell lines

**Figure 3:**
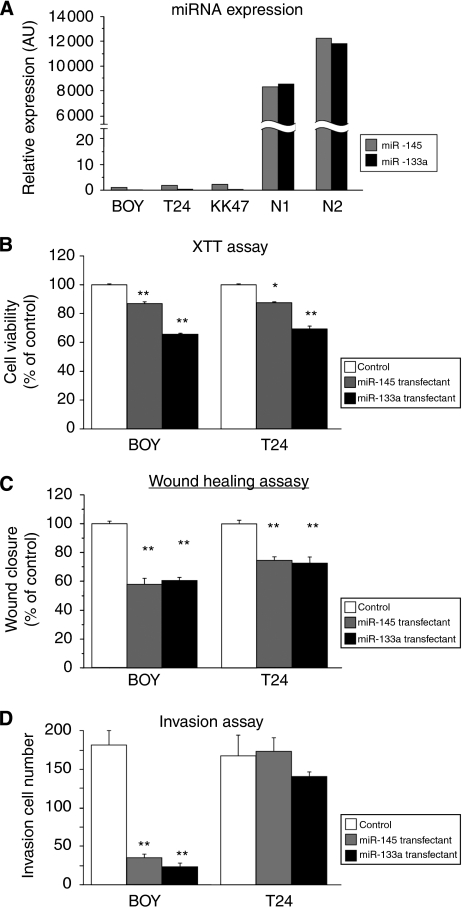
(**A**) *miR-145* and *miR-133a* expression in BC cell lines (BOY, T24, KK47) and normal human bladder mucosa (N1 and N2). (**B**–**D**) Effect of cell viabilities in *miR-145* and *miR-133a* transfectants: (**B**) cell growth determined by the XTT assay; (**C**) cell migration activity determined by the wound-healing assay; and (**D**) cell invasion activity determined by the matrigel invasion assay in BOY and T24 cell lines transfected with *miR-145* and *miR-133a*. ^*^P<0.005, ^**^P<0.0001.

The expression levels of *miR-145* and *miR-133a* were extremely low in the BC cell lines compared with normal bladder epithelia (N1 and N2) (Figure 3A), suggesting that endogenous *miR-145* or *miR-133a* in these cell lines does not affect cell viabilities. Therefore, we performed gain-of-function studies using the microRNA transfectants to investigate the functional role of *miR-145* and *miR-133a*. The XTT cell-growth assay showed significant cell-growth inhibitions in *miR-145* and *miR133a* transfectant compared with the controls from BOY and T24 cell lines (BOY, 86.6±1.6, 65.7±0.3, 100±0.6, respectively, *P*<0.0001; and T24, 87.4±0.6, 69.5±1.5, 100±0.9, respectively, *P*<0.0005; Figure 3B). The wound-healing assay showed significant cell migration inhibitions in *miR-145* and *miR133a* transfectant (BOY, 59.0±3.5, 58.1±3.4, 100.0±2.4, respectively, *P*<0.0001; and T24, 74.5±2.5, 72.3±4.0, 100.0±2.7, respectively, *P*<0.0001; Figure 3C). The matrigel invasion assay also showed significant cell invasion inhibitions in the transfectants compared with the control from the BOY cell lines (35.625±4.606, 24.000±4.516, 182.000±18.678, *P*<0.0001; Figure 3D). However, no significant difference was observed in the *miR-145* and *miR-133a* transfectants from T24 cell lines (173.875±16.607, 140.125±6.799, 167.000±27.367; Figure 3D). We did not subject KK47 cell line to these experiments because it showed a focal growth and it was not suitable for the experiments.

### Effect of FSCN1 knockdown on cell growth, invasion, and migration activity in BC cell lines

**Figure 4:**
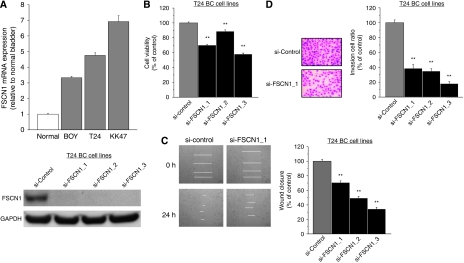
FSCN1-knockdown effect on BC cell viability by si-RNA: (**A**) upper, *FSCN1* mRNA expression in three BC cell lines (BOY, T24, KK47) by real-time RT–PCR; (**A**) lower, western blot revealed that FSCN1 protein was markedly decreased in three si-FSCN1 transfectants compared with the controls; (**B**) cell growth as revealed by the XTT assay; (**C**) cell migration activity by the wound-healing assay; and (**D**) cell invasion activity by the matrigel invasion assay in T24 cell lines transfected with si-FSCN1. si-FSCN1-transfected T24 cell lines exhibited a significant decrease in cell growth, migration, and invasion in comparison with the si-control transfectants. ^**^P<0.0001.

The expression levels of *FSCN1* mRNA were more than three-fold higher in BC cell lines than in the control (normal human bladder RNA). To examine the functional role of FSCN1, we performed loss of function studies using si-FSCN1-transfected T24 cell lines, which showed higher *FSCN1* mRNA expression levels compared with BOY (Figure 4A, upper). We did not subject KK47 cell line to these experiments because it showed a focal growth and it was not suitable for the experiments. FSCN1 protein expression was repressed by si-FSCN1 transfection (Figure 4A, lower). The XTT assay revealed significant cell-growth inhibition in the three si-FSCN1 transfectants in comparison with that in the si-control transfectant (% of cell viability; 69.9±1.3, 88.7±2.0, 58.0±1.4, and 100.0±1.3, respectively, *P*<0.0001; Figure 4B). The wound-healing assay also showed significant cell migration inhibitions in the si-FSCN1 transfectant compared with the counterparts (% of wound closure; 70.9±2.5, 49.4±2.5, 34.2±2.6, and 100.0±2.6, respectively, *P*<0.0001; Figure 4C). The matrigel invasion assay showed that the number of invading cell was significantly decreased in the si-FSCN1 transfectant compared with the counterparts (% of cell invasion; 39.0±4.6, 35.1±2.9, 18.3±2.5, and 100.0±3.9, respectively, *P*<0.0001; Figure 4D).

### Immunohistochemistry of FSCN1 and *in situ* hybridisation of *miR-145* in clinical BC samples

**Figure 5:**
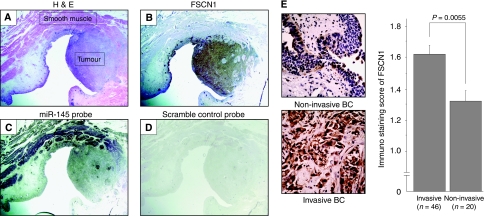
*In situ* hybridisation of *miR-145* and immunohistochemistry examination of FSCN1 in clinical BC specimens: (**A**) H&E staining, tumour, and surrounding smooth muscle; (**B**) immunohistochemical staining of FSCN1 showing strong expression in tumour lesion; (**C**) *in situ* hybridisation of *miR-145* showing faint expression in tumour lesion and strong expression in smooth muscle layer; (**D**) no staining by scramble-control probe; and (**E**) FSCN1 protein expression in invasive and non-invasive BC specimens. Low-grade bladder cancer without invasion (pTa) (upper panel, original magnification × 400). High-grade bladder cancer with involvement of the muscularis (pT2) (lower panel, original magnification × 400).

To visualise FSCN1 expression and the related microRNA expression in a tumour lesion and surrounding normal tissues, we performed immunohistochemistry of FSCN1 and *in situ* hybridisation of *miR-145* in FFPE tissues (Figure 5). H&E staining showed a high-grade tumour lesion surrounded by smooth muscle layers (Figure 5A). Immunohistochemistry revealed that FSCN1 was markedly expressed in the tumour lesion, whereas no expression was observed in adjacent tissues including the smooth muscle layers (Figure 5B). In contrast, *miR-145* was faintly expressed in the tumour lesion with the strong expression in the smooth muscle layers (Figure 5C). The scramble-control probe showed no significant staining in either the tumour or the smooth muscle layers (Figure 5D). Figure 5E shows immunostaining of FSCN1 in a non-invasive BC (pTa) and an invasive BC with involvement of the muscularis (pT2). There was faint staining in the non-invasive BC, whereas there was strong staining of cytosol and nuclei in the invasive BC. The staining score of the invasive BC (⩾pT1) was significantly higher than that of the non-invasive BC (pTa) (1.62±0.05 *vs* 1.33±0.07, *P*=0.0055). We found no correlation between FSCN1 expression and clinico-pathological parameters except for tumour stage.

## Discussion

Earlier studies showed that *miR-145* and *miR-133a* are commonly down-regulated in several human cancers and that their transfection reduces cell proliferation of each cancer cell line (Bandrés *et al*, 2006; Sempere *et al*, 2007; Slaby *et al*, 2007; Szafranska *et al*, 2007; Ozen *et al*, 2008; Schepeler *et al*, 2008; Wang *et al*, 2008, 2009a, 2009b; Wong *et al*, 2008a; Childs *et al*, 2009; Friedman *et al*, 2009; Liu *et al*, 2009; Takagi *et al*, 2009; Tong *et al*, 2009; Lin *et al*, 2009a). Consistent with earlier studies, we found significant cell-growth inhibitions in BC cell lines transfected with *miR-145* and *miR-133a* precursors. These results suggest that these microRNAs may have tumour suppressive functions through regulating oncogenic genes in human malignancies. Regarding BC, *miR-145* was listed in two of the three earlier studies investigating microRNA signatures in BC compared with normal control (Friedman *et al*, 2009; Lin *et al*, 2009a, 2009b). Moreover, this study is the first to show that *miR-133a* is a down-regulated microRNA in BC. An earlier study showed that *miR-133a* is abundantly expressed in muscle cells, and it may have a part in regulating proliferation and differentiation (McCarthy and Esser, 2007). Regarding the target genes, there are only three earlier studies showing that *miR-145* directly binds to c-Myc (Sachdeva *et al*, 2009) and insulin receptor substrate-1 (Shi *et al*, 2007), which are associated with cell proliferation and that *miR-133a*/*b* directly binds to pyruvate kinase type M2 expression, which is a potent oncogene in solid cancers (Wong *et al*, 2008b). Down-regulation of these microRNAs may have a critical function in BC development. Our cell invasion assay showed that there were significant decreases of invading cell number in the *miR-145* and *miR133a* transfectants from BOY, but not from T24 BC cell lines. These results suggest that another pathway might be more crucial than FSCN1 for invasiveness in some BCs. To find the target genes, web-based software was used in the earlier studies. However, the many candidate microRNAs identified by the web-based software often make it more difficult for researchers to find the crucial target genes. In this study, we used an oligo-microarray to screen the candidates from gene expression profiles in *miR-145* transfectant and found a new target gene, FSCN1, which was subsequently validated by the luciferase reporter assay. Thus, gene expression profiles from specific microRNA transfectant may be a good strategy for finding candidate genes targeted by microRNA.

FSCN1 functions in two major forms of actin-based structures: cortical cell protrusions that mediate interactions between cells and the extra-cellular matrix (ECM), cell-to-cell interactions, and cell migration; and cytoplasmic microfilamentous bundles that contribute to cell architecture and intracellular movements (Kureishy *et al*, 2002). The fascin–actin interaction is affected by extra-cellular cues, and certain ECM components induce bundling of actin by FSCN1 (Hashimoto *et al*, 2005). It is plausible that the activation of fascin through ECM substrates contributes to tumour growth, migration, and invasion. In BC, FSCN1 over-expression has been noted in three different immunohistochemistry studies (Tong *et al*, 2005; Karasavvidou *et al*, 2008; Soukup *et al*, 2008). Our immnohistochemical study consistently showed that the expression levels of FSCN1 were correlated with advanced tumour stage. In addition, tumour viability was markedly decreased in FSCN1-knockdown BC cell lines. These results strongly suggest that this molecule may function as an oncogene. It may be deeply associated with BC invasiveness and might be a useful staging biomarker for clinical BC.

Regarding FSCN1 regulation, several studies have reported that the actin-binding activity of fascin is inhibited by phosphorylation of residue Ser-39 by protein kinase C*α* (Adams *et al*, 1999); *β*-catenin/T cell factor signalling transactivates the FSCN1 promoter in human colon carcinoma cell lines (Vignjevic *et al*, 2007); and FSCN1 down-regulation is associated with a decrease in *β*-catenin and c-erbB-2 expression (Xie *et al*, 2005). However, to our knowledge, there has been no earlier study reporting the interaction between FSCN1 expression and particular microRNAs. We earlier reported that *miR-145* and *miR-133a* expressions are significantly down-regulated in BC tissue compared with normal bladder epithelium (Ichimi *et al*, 2009). In this study, we showed that *miR-145* and *miR-133a* directly target FSCN1, resulting in decreased *FSCN1* mRNA and its protein levels both *in vitro* and in clinical specimens. The question of how FSCN1 becomes over-expressed is still open, but one possible mechanism is through regulation by microRNAs. Loss of *miR-145* and *miR-133a*, both of which are endogenous FSCN1 inhibitors, may promote aberrant expression of FSCN1 contributing to pathogenesis and progression of BC.

In summary, through our microRNA profiling in BC, we have found that FSCN1 might have an oncogenic function in BC and *miR-145* and *miR-133a* might function as tumour suppressors through direct repression of FSCN1 in BC. As viral vector-mediated microRNA transduction might be applicable *in vivo* (Yang *et al*, 2006), our findings raise the possibility that *miR-145* and *miR-133a* may have potential therapeutic value in BC patients. In addition, FSCN1 may be a potential target for gene therapy of BC. As down-regulation of *miR-145* and *miR-133a* and over-expression of FSCN1 were commonly identified in various human malignancies, our findings may be crucial events in the development throughout human malignancies.

## Figures and Tables

**Table 1:** Patient characteristics

**Total number**	**66**
*Gender*	
Male	51
Female	15
	
*Age*	
Median age (range)	72 (47–92) years
	
*Stage*	
Superficial (pTa)	20
Invasive (⩾pT1)	46
	
*Grade*	
G1	7
G2	41
G3	18
	
*Operation*	
Cystectomy	17
TUR-BT	49
	
*Recurrence*	
Yes	38
No	28

Abbreviation: TUR-BT=transurethral resection of bladder tumour.

**Table 2:** Top 20 genes that were down-regulated by >0.5-fold in *miR-145* transfectants in comparison with the control

**Entrez gene ID**	**Gene symbol**	**Gene name**	**Log2 ratio**
6624	FSCN1	Fascin homologue 1, actin-bundling protein (*Strongylocentrotus purpuratus*)	−3.95
10447	FAM3C	Family with sequence similarity 3, member C	−3.26
203547	LOC203547	Hypothetical protein LOC203547	−3.17
2519	FUCA2	Fucosidase, *α*-L-2, plasma	−2.88
51280	GOLM1	Golgi membrane protein 1	−2.85
56674	TMEM9B	TMEM9 domain family, member B	−2.85
5094	PCBP2	Poly(rC)-binding protein 2	−2.81
84841	MGC15634	Hypothetical protein MGC15634	−2.80
2764	GMFB	Glia maturation factor, *β*	−2.63
91452	ACBD5	Acyl-coenzyme A-binding domain containing 5	−2.61
7048	TGFBR2	Transforming growth factor, *β* receptor II (70/80 kDa)	−2.57
8508	NIPSNAP1	Nipsnap homologue 1 (*Caenorhabditis elegans*)	−2.55
23075	SWAP70	SWAP-70 protein	−2.54
92675	DTD1	D-tyrosyl-tRNA deacylase 1 homologue (*Saccharomyces cerevisiae*)	−2.53
27250	PDCD4	Programmed cell death 4 (neoplastic transformation inhibitor)	−2.52
57552	AADACL1	Arylacetamide deacetylase-like 1	−2.49
4697	NDUFA4	NADH dehydrogenase (ubiquinone) 1 *α* subcomplex, 4, 9 kDa	−2.46
5530	PPP3CA	Protein phosphatase 3 (formerly 2B), catalytic subunit, *α* isoform	−2.39
51199	NIN	Ninein (GSK3B-interacting protein)	−2.26
89894	TMEM116	transmembrane protein 116	−2.03

Abbreviation: NADH=nicotinamide adenine dinucleotide.
